# 2523: Mentor training for KL2 Scholars through vertical integration

**DOI:** 10.1017/cts.2017.185

**Published:** 2018-05-10

**Authors:** Angela Merrifield, Michelle Lamere, Kelvin Lim, Megan Larson, David H. Ingbar

**Affiliations:** CTSI , University of Minnesota, Minneapolis, MN, USA

## Abstract

OBJECTIVES/SPECIFIC AIMS: The NIH states, “The training of the biomedical workforce has always been an integral part of the NIH mission… It takes just one good mentor to influence the career of a new investigator; it takes a robust culture of mentorship across the research community to strengthen, sustain and diversify the entire biomedical research enterprise.” The University of Minnesota’s CTSI-Education core strives to build and maintain a strong culture of mentoring by providing CTSI KL2 scholars an opportunity to mentor an undergraduate student participating in the Pathways to Research Program (PReP). Using this mentoring model, participants gain valuable benefits and CTSI’s culture of mentoring is strengthened. METHODS/STUDY POPULATION: Participating KL2 scholars are matched with a promising PReP scholar for a 12-week mentored research project. The PReP program selects top candidates through a highly competitive application process. Students work in their mentor’s lab full-time, funded by CTSI-Ed. They engage in additional activities together including a mentor/mentee, an interview activity and 2 social events. Junior faculty scholars are asked to participate as judges at CTSI’s Poster Session and are invited to present at PReP seminars. The program culminates with the announcement of the Junior Mentor of the Year, in which scholars nominate their mentors for the award. Junior faculty mentors receive support through a training course, Optimizing the Practice of Mentoring, mentor orientation and a roundtable discussion with the program director and other mentors. The program’s infrastructure is designed to foster mentee/mentor relationships through faculty and staff support. Junior faculty receive one-on-one coaching when faced with difficult mentoring situations and are recognized for their mentoring successes. RESULTS/ANTICIPATED RESULTS: Junior faculty mentors highly rate the program on the following points; the experience was a good use of time, I am satisfied with my experience, I would recommend this program to faculty colleagues and students. Undergraduates and Professional students rated their mentoring relationship as 1 of 3 best outcomes of the program. In exit surveys, their highly rated program successes include having a network that helps move their career forward, and confidence to persist through training to become a successful researcher. DISCUSSION/SIGNIFICANCE OF IMPACT: Creating a culture of mentoring is important to the strengthen, sustain and diversify the biomedical research workforce. This mentoring model contributes to the mission while vertically integrating CTSI-Ed’s KL2 and PReP programs. On an individual level, junior faculty improve communication and management skills, develop leadership qualities, increase their network, provide a sense of fulfilment and personal growth, and reinforce their own skills and knowledge of subject. They are also provided a top undergraduate student worker fully funded by the program.

